# Supplementary material for: Investigating the shared genetics of non-syndromic cleft lip/palate and facial morphology
Source: PLoS Genet. 2018 Aug 1;14(8):e1007501. doi: 10.1371/journal.pgen.1007501 (PMC6089455; doi:10.1371/journal.pgen.1007501)
Supplement: S1 Table — (DOCX) [file pgen.1007501.s001.docx]

**S1 Table.** Top GWAS hits for nsCL/P compared between published study and our meta-analysis

| **SNP** | **(Ludwig et al 2012) European**  **P-value** | **TDT + Bonn-II Meta-Analysis**  **P-value** |
| --- | --- | --- |
| rs560426 | 1.02 x 10^-6^ | 4.43 x 10^-5^ |
| rs861020 | 1.78 x 10^-6^ | 1.38 x 10^-5^ |
| rs987525 | 3.94 x 10^-34^ | 7.95 x 10^-20^ _1_ |
| rs7078160 | 2.81 x 10^-8^ | 3.99 x 10^-7^ |
| rs227731 | 4.26 x 10^-8^ | 4.50 x 10^-7^ |
| rs13041247 | 7.41 x 10^-4^ | 2.25 x 10^-3^ |
| rs742071 | 2.63 x 10^-7^ | 4.07 x 10^-6^ |
| rs7590268 | 4.05 x 10^-8^ | 2.17 x 10^-6^ |
| rs7632427 | 4.20 x 10^-5^ | 2.62 x 10^-4^ |
| rs12543318 | 1.02 x 10^-6^ | 1.49 x 10^-5^ |
| rs8001641 | 6.20 x 10^-10^ | 4.41 x 10^-8^ |
| rs1873147 | 2.81 x 10^-8^ | 4.22 x 10^-7^ |

_1_ rs987525 was removed in the trios in our QC hence the discrepancy in P values
