# Supplementary material for: Investigating the shared genetics of non-syndromic cleft lip/palate and facial morphology
Source: PLoS Genet. 2018 Aug 1;14(8):e1007501. doi: 10.1371/journal.pgen.1007501 (PMC6089455; doi:10.1371/journal.pgen.1007501)
Supplement: S2 Table — (DOCX) [file pgen.1007501.s002.docx]

**S2 Table.** Polygenic Transmission of nsCL/P genetic risk variants in independent European and Asian trios

| **P-value Inclusion Threshold** | **European Trios (N = 2209)** | | | **Asian Trios (N = 2593)** | | |
| --- | --- | --- | --- | --- | --- | --- |
|  | **Number of SNPs** | **Beta (95% C.I.)^1^** | **P Value** | **Number of SNPs** | **Beta (95% C.I.)^1^** | **P Value** |
| 0.000001 | 4 | 0.30 (0.23, 0.37) | 1.8x 10^-16^ | 10 | 0.12 (0.05, 0.19) | 8.0x 10^-4^ |
| 0.000005 | 13 | 0.30 (0.23, 0.37) | 1.1x 10^-16^ | 18 | 0.16 (0.09, 0.23) | 7.0x 10^-6^ |
| **0.00001** | **17** | **0.32 (0.25, 0.39)** | **3.5x 10^-18^** | **23** | **0.19 (0.12, 0.25)** | **1.7x 10^-7^** |
| 0.00005 | 44 | 0.25 (0.18, 0.33) | 1.0x 10^-11^ | 53 | 0.16 (0.09, 0.13) | 1.1x 10^-5^ |
| 0.0001 | 69 | 0.24 (0.17, 0.31) | 2.1x 10^-11^ | 81 | 0.12 (0.05, 0.19) | 0.001 |
| 0.0005 | 222 | 0.17 (0.10, 0.23) | 2.7x 10^-6^ | 244 | 0.06 (0.00, 0.13) | 0.056 |
| 0.001 | 405 | 0.15 (0.08, 0.22) | 4.6x 10^-5^ | 437 | 0.08 (0.01, 0.14) | 0.020 |
| 0.005 | 1,626 | 0.19 (0.12, 0.26) | 5.3x 10^-8^ | 1793 | 0.12 (0.05, 0.18) | 3.5x10^-4^ |
| 0.01 | 3,002 | 0.16 (0.09, 0.23) | 9.2x 10^-6^ | 3,334 | 0.10 (0.04, 0.17) | 0.002 |
| 0.05 | 11,400 | 0.16 (0.10, 0.23) | 1.7x 10^-6^ | 13,222 | 0.09 (0.02, 0.15) | 0.009 |
| 0.1 | 20,133 | 0.16 (0.10, 0.23) | 2.2x 10^-6^ | 23,421 | 0.06 (-0.01, 0.12) | 0.096 |
| 0.5 | 64,727 | 0.17 (0.10, 0.24) | 1.1x 10^-6^ | 74,832 | 0.03 (-0.03, 0.10) | 0.31 |
| 1 | 92,527 | 0.16 (0.09, 0.23) | 2.6x 10^-6^ | 107,809 | 0.03 (-0.04, 0.10) | 0.38 |

^1 Standardised difference in genetic score between parents and offspring^
