# Supplementary material for: Investigating the shared genetics of non-syndromic cleft lip/palate and facial morphology
Source: PLoS Genet. 2018 Aug 1;14(8):e1007501. doi: 10.1371/journal.pgen.1007501 (PMC6089455; doi:10.1371/journal.pgen.1007501)
Supplement: S3 Table — (DOCX) [file pgen.1007501.s003.docx]

**S3 Table.** nsCL/P Polygenic risk score SNPs

| **SNP** | **CHR:BP^1^** | **Effect Allele** | **Other Allele** | **nsCL/P Beta** | **nsCL/P S.E.** | **nsCL/P P-value** |
| --- | --- | --- | --- | --- | --- | --- |
| rs742071 | 1:18979874 | T | G | 0.2927 | 0.0573 | 3.3 x 10^-7^ |
| rs560426 | 1:94553438 | T | C | -0.2574 | 0.0564 | 5.1 x 10^-6^ |
| rs4147812 | 1:94575043 | A | C | 0.3234 | 0.0606 | 9.4 x 10^-8^ |
| rs12057415 | 1:94829769 | T | C | -0.2786 | 0.0572 | 1.1 x 10^-6^ |
| rs861020 | 1:209977111 | A | G | 0.3218 | 0.0660 | 1.1 x 10^-6^ |
| rs7590268 | 2:43540125 | T | G | -0.3428 | 0.0650 | 1.3 x 10^-7^ |
| rs1650504 | 5:158029550 | A | G | 0.2585 | 0.0584 | 9.5 x 10^-6^ |
| rs12543318 | 8:88868340 | A | C | -0.288 | 0.0594 | 1.3 x 10^-6^ |
| rs6470648 | 8:129716308 | A | G | 0.3081 | 0.0636 | 1.3 x 10^-6^ |
| rs11989880 | 8:129872982 | T | C | 0.4993 | 0.0586 | 1.5 x 10^-17^ |
| rs12548036 | 8:129947882 | T | G | 0.5416 | 0.0585 | 2.1 x 10^-20^ |
| rs1372452 | 8:130029034 | A | G | 0.5244 | 0.0778 | 1.6 x 10^-11^ |
| rs3138512 | 9:92222453 | A | G | 0.3085 | 0.0690 | 7.9 x 10^-6^ |
| rs4752028 | 10:118834991 | T | C | -0.4046 | 0.0703 | 8.8 x 10^-9^ |
| rs9545330 | 13:80699166 | A | G | 0.432 | 0.0684 | 2.6 x 10^-10^ |
| rs1258763 | 15:33050423 | T | C | 0.3049 | 0.0629 | 1.2 x 10^-6^ |
| rs1873147 | 15:63312632 | A | G | -0.3518 | 0.0621 | 1.4 x 10^-8^ |
| rs8076457 | 17:8943929 | T | C | 0.3175 | 0.0620 | 3.1 x 10^-7^ |
| rs227731 | 17:54773238 | T | G | -0.3148 | 0.0564 | 2.5 x 10^-8^ |
| rs1808191 | 17:62784028 | A | C | -0.3307 | 0.0707 | 2.9 x 10^-6^ |
| rs3746101 | 19:2050823 | T | G | 0.4474 | 0.0980 | 5.0 x 10^-6^ |

^1 CHR:BP – Chromosome and Base Pair Position on HG19^
