# Supplementary material for: Investigating the shared genetics of non-syndromic cleft lip/palate and facial morphology
Source: PLoS Genet. 2018 Aug 1;14(8):e1007501. doi: 10.1371/journal.pgen.1007501 (PMC6089455; doi:10.1371/journal.pgen.1007501)
Supplement: S4 Table — (DOCX) [file pgen.1007501.s004.docx]

**S4 Table.** Power calculations for polygenic risk scoring

| **3D facial Euclidean distances in ALSPAC**  **(N=3737)** | **Minimum Genetic Covariance** | **SNP heritability (h^2^) estimate from GCTA (95% C.I.)** | **Minimum Genetic Correlation between nsCL/P and developmental outcome detectable^1^**  **(95% C.I. from h^2^ estimates)** |
| --- | --- | --- | --- |
| Distance between subnasale and labiale superius  (Nasal-lip) | 0.045 | 0.34 (0.19, 0.49) | 0.17 (0.14, 0.23) |
| Distance between labiale inferius and pogonion  (Lip-chin) | 0.045 | 0.21 (0.06, 0.35) | 0.22 (0.17, 0.41) |
| Distance between left and right palpebrale inferius (Mid-point of eyes) | 0.045 | 0.34 (0.19, 0.49) | 0.17 (0.14, 0.23) |
| Distance between left and right alare (Nasal width) | 0.045 | 0.13 [0.00, 0.27) | 0.28 (0.19, 1] |
| Distance between labiales inferius and superius  (lip height) | 0.045 | 0.18 (0.03, 0.32) | 0.24 (0.18, 0.58) |
| Distance between left and right crista philtri  (philtrum width) | 0.045 | 0.20 (0.05, 0.35) | 0.23 (0.17, 0.45) |
| Distance between left and right cheilion  (lip width) | 0.045 | 0.18 (0.04, 0.33) | 0.24 (0.18, 0.50) |

**^1^** ${Genetic Correlation}_{AB}= \frac{Genetic Covariance_{AB}}{\sqrt{h_{A}^{2}h_{B}^{2}}}$
