# Supplementary material for: Investigating the shared genetics of non-syndromic cleft lip/palate and facial morphology
Source: PLoS Genet. 2018 Aug 1;14(8):e1007501. doi: 10.1371/journal.pgen.1007501 (PMC6089455; doi:10.1371/journal.pgen.1007501)
Supplement: S5 Table — (DOCX) [file pgen.1007501.s005.docx]

**S5 Table.** Independent philtrum width trait loci derived from the ALSPAC/3DFN summary statistics

| **Variant** | **CHR:BP** | **Effect allele / Other allele** | **ALSPAC (N=3707)** | | **3DFN (N=2429)** | | **Combined meta-analysis (N=6136)** | |
| --- | --- | --- | --- | --- | --- | --- | --- | --- |
|  |  |  | **Beta (95% C.I.)** | **P-value** | **Beta (95% C.I.)** | **P-value** | **Beta (95% C.I.)** | **P-value** |
| rs255877 | 5:112753584 | G/A | 0.24 (0.15, 0.32) | 6.2x10^-8^ | 0.16 (0.07, 0.25) | 8.1x10^-4^ | 0.20 (0.14, 0.26) | 3.8x10^-10^ |
| rs2522825 | 7:27111994 | T/C | -0.30 (-0.39, -0.20) | 2.8x10^-10^ | -0.08 (-0.17, 0.02) | 0.11 | -0.19 (-0.26, -0.13) | 1.4x10^-8^ |
