# Supplementary material for: Investigating the shared genetics of non-syndromic cleft lip/palate and facial morphology
Source: PLoS Genet. 2018 Aug 1;14(8):e1007501. doi: 10.1371/journal.pgen.1007501 (PMC6089455; doi:10.1371/journal.pgen.1007501)
Supplement: S6 Table — (DOCX) [file pgen.1007501.s006.docx]

**S6 Table.** Philtrum width associated SNPs in GTex

| **SNP (Effect allele)** | **Gene** | **Effect size of association with gene expression (P-value)** | **Tissue** |
| --- | --- | --- | --- |
| rs255877 (G) | *YTHDC2* | -0.32 (3.4x10^-9^)  -0.11 (2.1x10^-6^)  -0.14 (2.4x10^-5^) | Brain – Cerebellum  Thyroid  Lung |
|  | *CTD-2201G3.1* | 0.23 (3.6x10^-5^) | Skin – sun exposed (lower leg) |
| rs2522825 (T) | *SKAP2* | 0.20 (3.2x10^-14^) | Whole blood |
|  | *HOXA4* | 0.43 (2.2x10^-9^) | Whole blood |
|  | *HOXA-AS2* | 0.41 (7.0x10^-9^) | Whole blood |
|  | *HOTAIRM1* | 0.35 (3.1x10^-8^) | Whole blood |
|  | *HOXA2* | 0.37 (4.4x10^-8^) | Whole blood |
|  | *HOXA5* | 0.31 (4.0x10^-6^) | Skin – not sun exposed (suprapubic) |
|  | *HOXA1* | 0.33 (1.2x10^-5^)  0.24 (2.1x10^-5^) | Whole blood  Skin – sun exposed (lower leg) |
|  | *HOXA6* | 0.24 (4.3x10^-5^) | Skin – not sun exposed (suprapubic) |
