# Supplementary material for: Investigating the shared genetics of non-syndromic cleft lip/palate and facial morphology
Source: PLoS Genet. 2018 Aug 1;14(8):e1007501. doi: 10.1371/journal.pgen.1007501 (PMC6089455; doi:10.1371/journal.pgen.1007501)
Supplement: S7 Table — (DOCX) [file pgen.1007501.s007.docx]

**S7 Table.** Proxy SNPs (for philtrum width associated variants) in nsCL/P summary statistics

| **SNP** | **Proxy SNP / 1000G CEU & GBR r^2^** | **Proxy CHR:BP** | **Effect allele / Other allele** | **Philtrum width Beta** | **Philtrum width S.E.** | **nsCL/P Beta** | **nsCL/P S.E.** |
| --- | --- | --- | --- | --- | --- | --- | --- |
| rs255877 | rs13188946 / 0.97 | 5:112722855 | T/C | 0.20 | 0.032 | 0.0088 | 0.058 |
| rs2522825 | rs2712248 / 0.95 | 7:27120689 | T/C | -0.18 | 0.039 | -0.11 | 0.061 |
