# Supplementary material for: Investigating the shared genetics of non-syndromic cleft lip/palate and facial morphology
Source: PLoS Genet. 2018 Aug 1;14(8):e1007501. doi: 10.1371/journal.pgen.1007501 (PMC6089455; doi:10.1371/journal.pgen.1007501)
Supplement: S8 Table — (DOCX) [file pgen.1007501.s008.docx]

**S8 Table.** Biologically plausible facial phenotypes

| **Facial phenotype** | **Justification for inclusion** |
| --- | --- |
| Nasal lip height | Relevance to cleft lip |
| Lip chin height | Weinberg et al (Orthodontics & craniofacial research 2009) – compared phenotype between parents of nsCL/P children and control parents. |
| Inter-palpebrale width (mid-point of eyes) | Weinberg et al (Orthodontics & craniofacial research 2009) – compared phenotype between parents of nsCL/P children and control parents. |
| Nasal width | Boehringer et al (EJHG 2011) – looked for association between phenotype and nsCL/P SNPs. |
| Lip height | Relevance to cleft lip |
| Philtrum width | Relevance to cleft lip |
| Lip width | Relevance to cleft lip |
