# Supplementary material for: Investigating the shared genetics of non-syndromic cleft lip/palate and facial morphology
Source: PLoS Genet. 2018 Aug 1;14(8):e1007501. doi: 10.1371/journal.pgen.1007501 (PMC6089455; doi:10.1371/journal.pgen.1007501)
Supplement: S9 Table — (DOCX) [file pgen.1007501.s009.docx]

**S9 Table.** Parameters in Polygenic Risk Score analysis power calculations

| **Parameter** | **Value** | **Source** |
| --- | --- | --- |
| Sample size of training sample | 3987 | determined from data |
| Prevalence of nsCL/P in training sample | 0.305 | determined from data |
| Population prevalence of nsCL/P | 0.001 | IPDTOC Working Group. "Prevalence at birth of cleft lip with or without cleft palate: data from the International Perinatal Database of Typical Oral Clefts (IPDTOC)." (2011). |
| Number of independent SNPs common to both arrays (r^2^<0.1) | 75,737 | determined from data |
| h^2^ of nsCL/P | 0.2 | AVENGEME estimate |
| Proportion of null markers | 0.992 | AVENGEME estimate |
