# Supplementary material for: Investigating the shared genetics of non-syndromic cleft lip/palate and facial morphology
Source: PLoS Genet. 2018 Aug 1;14(8):e1007501. doi: 10.1371/journal.pgen.1007501 (PMC6089455; doi:10.1371/journal.pgen.1007501)
Supplement: S10 Table — (DOCX) [file pgen.1007501.s010.docx]

**S10 Table.** nsCL/P Mendelian randomization SNPs

| **SNP** | **CHR:BP^1^** | **Effect Allele / Other Allele** | **nsCL/P Beta** | **nsCL/P S.E.** | **Philtrum width Beta** | **Philtrum width S.E.** |
| --- | --- | --- | --- | --- | --- | --- |
| rs7590268 | 2:43540125 | T/G | -0.328 | 0.065 | 0.05367 | 0.05052 |
| rs987525 | 8:129946154 | A/C | 0.8286 | 0.0909 | -0.1045 | 0.05173 |
| rs7078160 | 10:118827560 | A/G | 0.3996 | 0.0704 | 0.0806 | 0.05723 |
| rs8001641 | 13:80692811 | A/G | 0.3573 | 0.0581 | -0.002105 | 0.04324 |
| rs1873147 | 15:63312632 | A/G | -0.3518 | 0.0621 | 0.04914 | 0.04818 |
| rs227731 | 17:54773238 | T/G | -0.3148 | 0.0564 | 0.09291 | 0.04341 |

^1 CHR:BP – Chromosome and Base Pair Position on HG19^
